# Experimental measurements and modelling of viscosity and density of calcium and potassium chlorides ternary solutions

**DOI:** 10.1038/s41598-020-73484-4

**Published:** 2020-10-01

**Authors:** Mohammad Arshad, Ahmed Easa, Hazim Qiblawey, Mustafa Nasser, Abdelbaki Benamor, Rahul Bhosale, Mohammad Al-Ghouti

**Affiliations:** 1 Department of Chemical Engineering, College of Engineering, Qatar University, Doha, Qatar; 2 Central Laboratory Unit, Qatar University, Doha, Qatar; 3 Gas Processing Centre, College of Engineering, Qatar University, Doha, Qatar; 4 Department of Biological and Environmental Sciences, College of Arts and Sciences, Qatar University, Doha, Qatar

**Keywords:** Chemical engineering, Mineralogy

## Abstract

Measured viscosity and density data for ternary aqueous solutions of CaCl_2_ and KCl are presented at temperatures between 293 and 323 K with 5 K increment. A modified Jones–Dole was introduced by adding extra terms and proved to be suitable for modelling of the viscosity data. Goldsack and Franchetto, Hu and Exponential models are used to correlate the viscosity data, too. Al models are correlated as a function of temperature and concentration. All models had successfully predicted the viscosity with high precision reaching a maximum average absolute deviation (AAD) of less than 2.3%. The modified Jones–Dole showed the best results among other models. Viscosity of the ternary solution is higher than the viscosity of water by about 15% at low concentrations and reaches about 270% at the highest concentrations. The amount of CaCl_2_ has more significant effect on the ternary mixture viscosity compared to KCl. This has created difficulty in measuring the viscosity and consequently the challenge in finding the different models parameters. Ternary solution densities were successfully correlate with Kumar’s model with AAD of less than 0.4%. Comparison of the ternary solution density and viscosity with the few available data literature showed a good agreement.

## Introduction

Transport properties data of electrolyte solution is required for design and operation of many industrial processes like crystallization, food processing and fertilizer production. Dynamic viscosity is one of the important thermo-physical properties. The measurement of aqueous solution viscosity is expensive and time-consuming, especially when more than one electrolyte is involved. Theoretical and experimental investigations of viscosity of electrolyte aqueous mixture have been subject of interest to many researchers^1–5^.

Reaching clean water resources is becoming more difficult due to the increase of population, urbanization and climate changes. Nontraditional water resources are needed in order to cover the human’s need of water. In the past two decades, desalination technology was practiced and proved to be a sustainable solution to fill this gap^6,7^. In desalination, feed of saline water with high concentrations of electrolyte solutions are used to get the fresh water. Energy is needed to separate the salts from water to get the fresh water stream. In addition to the fresh water stream, another by-product stream is generated with substantially higher electrolytes concentration than the feed. This stream is called concentrate or brine stream. Seawater is usually the feed stream and the brine disposal is returned back to the sea^8^. Due to more strict environmental regulations in returning the brine stream back to the see, new technologies are required to mitigate this impact^9^. Fractional crystallization is among them, where the different salts in seawater including sodium chloride, potassium chloride, magnesium chloride and calcium chloride are crystallized as solid crystal and sold as high quality salts in the market. Brine streams are introduced to evaporators to increase the concentration of the salts to a level where crystallization takes place in the crystallizers. Depending on salts solubility, crystallization will take place. Therefore, NaCl will crystallize first leaving the brine concentrated with the rest of the other salts to leave to the next crystallization stage and so on^10,11^.

Concentrate disposal with high concentration of electrolyte content of both KCl and CaCl_2_ among them is not only limited to desalination processes^12^. Other industries sharing this stream are mining processes such as oil and potash industries, salt dome for the storage of hydrocarbons and rejected brine from solar ponds used for heat generation^13–15^. In this work, the concentrated stream which contains both KCl and CaCl_2_ will be investigated for its viscosity and density.

To calculate dimensionless quantities like Reynolds, Schmidt and Sherwood numbers, which they are relevant to engineering design applications, viscosity and density data are required. Therefore, both viscosity and density are closely related and are of importance to estimate the pumping cost, pipeline sizing and design of the evaporators and crystallizers in the brine recovery process^16,17^.

Viscosity and density data for CaCl_2_ + KCl + H_2_O is available only at 298 K^18^. In this work, viscosity and density data for CaCl_2_ + KCl + H_2_O at temperature from 293.15 to 323.15 K are presented. The data were correlated using three known models in the literature and one modified equation. Models are developed as function of both concentration and temperature since most of the related industrial processes are running under a medium- or low-pressure environment. To the best of our knowledge such data have not been reported in the literature before.

## Experimental

The used chemicals in this work are listed in Table 1. All chemicals were used without further purification. Procedure used in this work is similar to the one followed in the previous work^18^. Briefly, stock solutions were prepared by dissolving weighed amounts of CaCl_2_ and KCl in double distilled water with a resistivity of 18.2 mΩ cm under continuous stirring. Viscosity and density are measured for ternary solutions prepared from the stock solutions by dilution. A precise analytical balance with an accuracy of 0.0001 g from Kern (ABS220-4) was used to prepare all the ternary solutions (gravimetric method). Ternary solutions were left under gentle stirring for a while to be sure that there is no crystal formation and they stay clear. Appearance of any crystals is a sign that the mixture is supersaturated and the sample was excluded because supersaturation was reached. This was considered as the maximum concentration in this study. In all samples, water was supplied from a Millipore water system. Each salt in the prepared ternary solutions were denoted as i = 1 for KCl, i = 2 for CaCl_2_ and i = 3 for H_2_O.

**Table 1:** Chemicals description.

Chemical name	Sources	Initial mass fraction purity	Purification method
Calcium chloride dihydrate	BDH	0.99	None
Potassium chloride	Fischer scientific	0.995	None

Calibrated Ubbelhode viscometer with a capillary diameter of 0.00053 m (capillary type 0a) from SCHOTT was used to measure the kinematic viscosities. The viscometer has been further calibrated with double de-ionized water. The transit time of the liquid meniscus through the capillary of the viscometer using stopwatch was used to measure the kinematic viscosity of a given solution. The time was measured with a precision of ± 0.01 s. Each measurement was repeated five times to ensure reproducibility of the data. A maximum deviation of 0.4% was observed in the measurements. Viscosity was measured within a temperature range between 293.15 and 323.15 K, with increment of 5 K. The range of the studied molality was between 0.5 and 4 mol kg^−1^. Densities of the solutions were measured precisely using an Anton Paar DMA 4500M density meter with an oscillating U-tube sensor. Double de-ionized water and toluene were used for calibration purposes. The uncertainty in temperature measurements is estimated to be 0.02 K (k = 1).

Solutions prepared for viscosity measurements were used to determine their densities. The density measurement accuracy was 0.00005 g cm^−3^ and repeatability was 0.00001 g cm^−3^. Equation (1) was used to calculate the dynamic viscosity;

1 $$ \frac{\eta }{\rho } = kt $$

*k* represents the constant of the viscometer provided by the manufacturer and *t* is the flow time in seconds. The temperature was controlled with a thermostated water circulator. The precision was ± 0.01 K. The dynamic viscosity measurements had uncertainty of 0.003 mPa s.

## Results and discussion

### Experimental data

#### Comparison of experimental data with published data

As mentioned, viscosity and density data of this ternary system are only available at 298 K. Very few studies were found to have the same concentration in order to be compared with the current study. Zhang et al.^19^ presented closer concentrations. It was found that deviations are reasonable. Maximum deviation was found to be 5.8% for viscosity at m_1_ = 3.0 mol kg^−1^ and m_2_ = 1.0 mol kg^−1^, other viscosity data showed deviations around 1%. Density values are closer to the published data with a maximum deviation 0.49% as density is straightforward function of concentrations. Comparison of densities and viscosities is presented in Table 2.

**Table 2:** Comparison of experimental data with the published data^19^.

m_1_ (mol kg^−1^)	m_2_ (mol kg^−1^)	Density (g cm^−3^)			Viscosity (mPa s)		
		This work	Literature	Deviation (%)	This work	Literature	Deviation (%)
3.0254	1.0033	1.1880	1.1886	0.0505	1.3165	1.2438	5.8450
1.5049	1.5049	1.1733	1.1739	0.0511	1.4249	1.4015	1.6696
2.0672	2.0672	1.2224	1.2285	0.4965	1.6944	1.7120	1.0280
0.5161	0.5163	1.0620	1.0640	0.1880	1.0495	1.0305	1.8438
1.0008	1.0013	1.1195	1.1206	0.0982	1.1975	1.1905	0.5880
			AAD	0.1769		AAD	2.1949

#### Discussion on experimental data

Densities and viscosities of the ternary aqueous solutions of KCl and CaCl_2_ are presented in Tables 3, 4, 5, 6, 7, 8 and 9 at the investigated molalities between 0.5 and 4 mol kg^−1^. Analyzing the density data, it was found that both solutes contributes positively to the density as expected. Analyze the effect of both separately reveals that CaCl_2_ has more significant effect on the density of the mixture. For example, referring to Table 3, increase in molality of KCl (*m*_1_) from 0.5 to 1.0 mol kg^−1^ increases the density by 1.9%. On the other hand, same increase in molality of CaCl_2_ increases the density by 3.80%. However, variation in viscosity depends on the solute molalities (m_1_ and m_2_) and does not follow one trend. Jones–Dole equation defined below helps in understanding trend of viscosity of electrolytes aqueous solution.

2 $$ \frac{\eta }{{\eta_{0} }} = 1 + Am^{1/2} + Bm $$

where m is the ion concentration in mol kg^−1^, η is the viscosity of the solution, and η_0_ is the viscosity of pure solvent. A is Falkenhagen coefficient, which is determined by ion–ion interactions and may be calculated analytically by Debye–Hückel theory. The B-coefficient is related to the strength of ion–solvent interactions. Ions with positive B-coefficients are classified as kosmotropes (structure maker) and negative B-coefficients as chaotropes (structure breaker). A structure make ion (+ve B value) is supposed to increase the viscosity while structure breaker (−ve value of B) will decrease the viscosity.

**Table 3:** Experimental and calculated densities $\rho$ and viscosities $\eta$ at T = 293.15 K for KCl (1) + CaCl_2_ (2) + H_2_O (3) system.

*m*_1_ (mol kg^−1^)	Experimental data		Calculated viscosity (mPa s)		Predicted density
	$\rho$ (g cm^−3^)	$\eta$ (mPa s)	Equation (5)	Hu’s model	$\rho$ (g cm^−3^)
			*m*_2_ = 0.5		
0.5	1.0635	1.1645	1.1455	1.1377	1.0609
1.0	1.0838	1.1614	1.1484	1.1425	1.0811
1.5	1.1031	1.1583	1.1553	1.1508	1.1005
2.0	1.1216	1.1679	1.1657	1.1616	1.1190
2.5	1.1435	1.1841	1.1796	1.1749	1.1369
3.0	1.1565	1.1926	1.1976	1.1917	1.1540
3.5	1.1726	1.2045	1.2165	1.2092	1.1704
			*m*_2_ = 1.0		
0.5	1.1035	1.3347	1.3231	1.3307	1.1024
1.0	1.1217	1.3284	1.3344	1.3426	1.1214
1.5	1.1409	1.3310	1.3491	1.3563	1.1395
2.0	1.1576	1.3500	1.3703	1.3754	1.1572
2.5	1.1744	1.3782	1.3902	1.3924	1.1735
3.0	1.1903	1.3862	1.4157	1.4148	1.1895
			*m*_2_ = 1.5		
0.5	1.1406	1.5371	1.5378	1.5551	1.1426
1.0	1.1583	1.5604	1.5577	1.5727	1.1600
1.5	1.1755	1.5799	1.5803	1.5925	1.1766
2.0	1.1918	1.5867	1.6080	1.6171	1.1928
2.5	1.2076	1.6007	1.6388	1.6449	1.2083
			*m*_2_ = 2.0		
0.5	1.1775	1.8036	1.8004	1.8123	1.1796
1.0	1.1933	1.8231	1.8283	1.8394	1.1957
1.5	1.2094	1.8428	1.8596	1.8705	1.2111
2.0	1.2249	1.8654	1.8987	1.9099	1.2265
			*m*_2_ = 2.5		
0.5	1.2117	2.1489	2.1225	2.1234	1.2132
1.0	1.2270	2.1653	2.1583	2.1657	1.2282
1.5	1.2423	2.1989	2.1986	2.2136	1.2430
			*m*_2_ = 3.0		
0.5	1.2439	2.5305	2.5226	2.5215	1.2440
1.0	1.2586	2.5733	2.5661	2.5842	1.2585
			*m*_2_ = 3.5		
0.5	1.2740	3.0547	3.0233	3.0405	1.2729
0.1	1.2888	3.1415	3.0761	3.1306	1.2875
			m_2_ = 4.0		
0.5	1.3038	3.7359	3.6613	3.7183	1.3012
			AAD% = 0.97	AAD% = 1.07	AAD% = 0.13
			SD = 0.0255	SD = 0.0201	SD = 0.0020

The standard uncertainties (u) are u(T) = 0.02 K, u(m) = 1.0 × 10^−4^ mol kg^−1^. The combined expanded uncertainty (U_c_) are U_c_(ρ) = 5.0 × 10^−5^ g cm^−3^ (0.95 level of confidence) and U_c_($\eta$) = 0.003 mPa s (0.95 level of confidence).

**Table 4:** Experimental and calculated densities $\rho$ and viscosities $\eta$ at T = 298.15 K for KCl (1) + CaCl_2_ (2) + H_2_O (3) system.

*m*_1_ (mol kg^−1^)	Experimental data		Calculated viscosity (mPa s)		Predicted density
	$\rho$ (g cm^−3^)	$\eta$ (mPa s)	Equation (5)	Hu’s model	$\rho$ (g cm^−3^)
			*m*_2_ = 0.5		
0.5	1.0620	1.0495	1.0279	1.0224	1.0622
1.0	1.0819	1.0496	1.0353	1.0304	1.0818
1.5	1.1013	1.0497	1.0453	1.0407	1.1006
2.0	1.1197	1.0622	1.0578	1.0525	1.1186
2.5	1.1414	1.0738	1.0727	1.0658	1.1360
3.0	1.1544	1.0817	1.0910	1.0810	1.1530
3.5	1.1704	1.0991	1.1099	1.0956	1.1694
			*m*_2_ = 1.0		
0.5	1.1017	1.1979	1.1891	1.1974	1.1008
1.0	1.1195	1.1975	1.2043	1.2095	1.1192
1.5	1.1388	1.2134	1.2215	1.2222	1.1369
2.0	1.1555	1.2221	1.2438	1.2382	1.1546
2.5	1.1722	1.2692	1.2644	1.2509	1.1712
3.0	1.1880	1.3165	1.2895	1.2663	1.1878
			*m*_2_ = 1.5		
0.5	1.1387	1.3839	1.3829	1.3949	1.1381
1.0	1.1562	1.4057	1.4059	1.4090	1.1559
1.5	1.1733	1.4249	1.4304	1.4230	1.1731
2.0	1.1895	1.4350	1.4587	1.4388	1.1902
2.5	1.2028	1.4773	1.4891	1.4546	1.2071
			*m*_2_ = 2.0		
0.5	1.1750	1.6218	1.6190	1.6196	1.1747
1.0	1.1911	1.6724	1.6494	1.6363	1.1921
1.5	1.2069	1.6691	1.6819	1.6532	1.2093
2.0	1.2224	1.6944	1.7205	1.6734	1.2269
			*m*_2_ = 2.5		
0.5	1.2093	1.9398	1.9082	1.8903	1.2100
1.0	1.2245	1.9457	1.9458	1.9105	1.2276
1.5	1.2397	1.9879	1.9865	1.9305	1.2453
			*m*_2_ = 3.0		
0.5	1.2414	2.3071	2.2666	2.2336	1.2439
1.0	1.2560	2.3096	2.3112	2.2556	1.2621
			*m*_2_ = 3.5		
0.5	1.2713	2.7324	2.7131	2.6738	1.2760
1.0	1.2851	2.7906	2.7663	2.6938	1.2956
			*m*_2_ = 4.0		
0.5	1.3010	3.3480	3.2776	3.2354	1.3068
			*AAD%* = 0.88	*AAD%* = 0.73	*AAD%* = 0.18
			*SD* = 0.0212	*SD* = 0.0134	*SD* = 0*.*0031

The standard uncertainties (*u*) are *u*(*T*) = 0.02 K, *u(m)* = 1.0 × 10^−4^ mol kg^−1^. The combined expanded uncertainty (*U*_*c*_) are *U*_*c*_(*ρ*) = 5.0 × 10^−5^ g cm^−3^ (0.95 level of confidence) and U_c_($\eta$) = 0.003 mPa s (0.95 level of confidence).

**Table 5:** Experimental and calculated densities $\rho$ and viscosities $\eta$ at T = 303.15 K for KCl (1) + CaCl_2_ (2) + H_2_O (3) system.

*m*_1_ (mol kg^−1^)	Experimental data		Calculated viscosity (mPa s)		Predicted density
	$\rho$ (g cm^−3^)	$\eta$ (mPa s)	Equation (5)	Hu’s model	$\rho$ (g cm^−3^)
			*m*_2_ = 0*.*5		
0.5	1.0601	0.9490	0.9283	0.9204	1.0601
1.0	1.0800	0.9466	0.9378	0.9299	1.0797
1.5	1.0993	0.9576	0.9491	0.9411	1.0985
2.0	1.1176	0.9710	0.9619	0.9531	1.1165
2.5	1.1393	0.9752	0.9765	0.9664	1.1339
3.0	1.1521	0.9880	0.9935	0.9820	1.1508
3.5	1.1681	1.0089	1.0105	0.9978	1.1669
			*m*_2_ = 1.0		
0.5	1.0998	1.0868	1.0763	1.0783	1.0989
1.0	1.1172	1.0875	1.0916	1.0920	1.1172
1.5	1.1367	1.0943	1.1083	1.1064	1.1349
2.0	1.1533	1.1129	1.1289	1.1247	1.1523
2.5	1.1699	1.1420	1.1474	1.1410	1.1684
3.0	1.1857	1.1505	1.1694	1.1616	1.1845
			*m*_2_ = 1.5		
0.5	1.1366	1.2637	1.2534	1.2622	1.1361
1.0	1.1539	1.2738	1.2747	1.2801	1.1535
1.5	1.1711	1.2823	1.2967	1.2994	1.1702
2.0	1.1871	1.3095	1.3217	1.3227	1.1866
2.5	1.2028	1.3256	1.3479	1.3488	1.2025
			*m*_2_ = 2.0		
0.5	1.1727	1.4665	1.4679	1.4739	1.1721
1.0	1.1887	1.5057	1.4946	1.4998	1.1886
1.5	1.2045	1.5133	1.5228	1.5292	1.2047
2.0	1.2200	1.5328	1.5559	1.5659	1.2207
			*m*_2_ = 2.5		
0.5	1.2069	1.7343	1.7285	1.7290	1.2064
1.0	1.2221	1.7514	1.7607	1.7683	1.2223
1.5	1.2372	1.8023	1.7951	1.8131	1.2380
			*m*_2_ = 3.0		
0.5	1.2389	2.0640	2.0491	2.0516	1.2391
1.0	1.2534	2.0813	2.0865	2.1094	1.2547
			*m*_2_ = 3.5		
0.5	1.2687	2.4762	2.4454	2.4653	1.2700
1.0	1.2832	2.5279	2.4894	2.5482	1.2858
			*m*_2_ = 4.0		
0.5	1.2982	3.0180	2.9431	2.9970	1.2996
			*AAD%* = 0.90	*AAD%* = 0.99	*AAD%* = 0.120
			*SD* = 0.0202	*SD* = 0.0155	*SD* = 0*.*0018

The standard uncertainties (*u*) are *u*(*T*) = 0.02 K, *u(m)* = 1.0 × 10^−4^ mol kg^−1^. The combined expanded uncertainty (*U*_*c*_) are *U*_*c*_(*ρ*) = 5.0 × 10^−5^ g cm^−3^ (0.95 level of confidence) and U_c_($\eta$) = 0.003 mPa s (0.95 level of confidence).

**Table 6:** Experimental and calculated densities $\rho$ and viscosities $\eta$ at T = 308.15 K for KCl (1) + CaCl_2_ (2) + H_2_O (3) system.

*m*_1_ (mol kg^−1^)	Experimental data		Calculated viscosity (mPa s)		Predicted viscosity
	$\rho$ (g cm^−3^)	$\eta$ (mPa s)	Equation (5)	Hu’s Model	$\rho$ (g cm^−3^)
			*m*_2_ = 0.5		
0.5	1.0582	0.8696	0.8427	0.8491	1.0587
1.0	1.0778	0.8657	0.8511	0.8572	1.0785
1.5	1.0972	0.8764	0.8604	0.8672	1.0972
2.0	1.1155	0.8835	0.8706	0.8783	1.1150
2.5	1.1370	0.8957	0.8821	0.8905	1.1322
3.0	1.1498	0.9097	0.8957	0.9043	1.1488
3.5	1.1658	0.9305	0.9099	0.9174	1.1649
			*m*_2_ = 1.0		
0.5	1.0977	0.9649	0.9809	0.9900	1.0976
1.0	1.1149	0.9927	0.9919	1.0017	1.1155
1.5	1.1345	1.0043	1.0034	1.0141	1.1328
2.0	1.1510	1.0214	1.0180	1.0293	1.1499
2.5	1.1676	1.0244	1.0305	1.0416	1.1662
3.0	1.1833	1.0579	1.0462	1.0560	1.1825
			*m*_2_ = 1.5		
0.5	1.1344	1.1488	1.1478	1.1534	1.1329
1.0	1.1515	1.1630	1.1616	1.1686	1.1502
1.5	1.1687	1.1766	1.1754	1.1835	1.1671
2.0	1.1847	1.2012	1.1914	1.1996	1.1841
2.5	1.2003	1.2145	1.2082	1.2154	1.2010
			*m*_2_ = 2.0		
0.5	1.1705	1.3391	1.3510	1.3514	1.1675
1.0	1.1864	1.3776	1.3672	1.3695	1.1849
1.5	1.2020	1.3752	1.3840	1.3870	1.2023
2.0	1.2174	1.4078	1.4048	1.4071	1.2203
			*m*_2_ = 2.5		
0.5	1.2044	1.5783	1.5983	1.5957	1.2021
1.0	1.2195	1.6039	1.6169	1.6157	1.2203
1.5	1.2346	1.6433	1.6367	1.6349	1.2388
			*m*_2_ = 3.0		
0.5	1.2363	1.8760	1.9020	1.8999	1.2369
1.0	1.2508	1.8966	1.9226	1.9194	1.2562
			*m*_2_ = 3.5		
0.5	1.2660	2.2371	2.2751	2.2743	1.2708
1.0	1.2805	2.3077	2.2991	2.2911	1.2920
			*m*_2_ = 4.0		
0.5	1.2954	2.7386	2.7390	2.7300	1.3033
			*AAD%* = 0.9558	*AAD%* = 0.87	*AAD%* = 0.18
			*SD* = 0.0151	*SD* = 0.0139	*SD* = 0.0031

The standard uncertainties (*u*) are *u*(*T*) = 0.02 K, *u(m)* = 1.0 × 10^−4^ mol kg^−1^. The combined expanded uncertainty (*U*_*c*_) are *U*_*c*_(*ρ*) = 5.0 × 10^−5^ g cm^−3^ (0.95 level of confidence) and U_c_($\eta$) = 0.003 mPa s (0.95 level of confidence).

**Table 7:** Experimental and calculated densities $\rho$ and viscosities $\eta$ at T = 313.15 K for KCl (1) + CaCl_2_ (2) + H_2_O (3) system.

*m*_1_ (mol kg^−1^)	Experimental data		Calculated viscosity (mPa s)		Predicted density
	$\rho$ (g cm^−3^)	$\eta$ (mPa s)	Equation (5)	Hu’s model	$\rho$ (g cm^−3^)
			*m*_2_ = 0*.*5		
0.5	1.0561	0.7879	0.7713	0.7675	1.0561
1.0	1.0756	0.7920	0.7836	0.7793	1.0757
1.5	1.0950	0.8051	0.7966	0.7922	1.0945
2.0	1.1132	0.8145	0.8102	0.8059	1.1124
2.5	1.1347	0.8270	0.8248	0.8206	1.1296
3.0	1.1474	0.8396	0.8412	0.8372	1.1461
3.5	1.1634	0.8634	0.8576	0.8540	1.1617
			*m*_2_ = 1.0		
0.5	1.0953	0.9059	0.8970	0.8997	1.0947
1.0	1.1125	0.9108	0.9137	0.9148	1.1128
1.5	1.1322	0.9237	0.9306	0.9306	1.1300
2.0	1.1486	0.9410	0.9501	0.9497	1.1467
2.5	1.1652	0.9698	0.9675	0.9672	1.1620
3.0	1.1808	0.9797	0.9876	0.9883	1.1769
			*m*_2_ = 1.5		
0.5	1.1321	1.0509	1.0458	1.0513	1.1305
1.0	1.1492	1.0659	1.0669	1.0707	1.1471
1.5	1.1663	1.0809	1.0879	1.0914	1.1627
2.0	1.1823	1.1011	1.1105	1.1154	1.1777
2.5	1.1978	1.1173	1.1338	1.1417	1.1917
			*m*_2_ = 2.0		
0.5	1.1681	1.2273	1.2245	1.2279	1.1643
1.0	1.1839	1.2598	1.2497	1.2547	1.1793
1.5	1.1995	1.2684	1.2752	1.2844	1.1934
2.0	1.2148	1.2979	1.3041	1.3201	1.2069
			*m*_2_ = 2.5		
0.5	1.2019	1.4444	1.4400	1.4417	1.1963
1.0	1.2170	1.4731	1.4693	1.4800	1.2097
1.5	1.2320	1.4963	1.4995	1.5227	1.2222
			*m*_2_ = 3.0		
0.5	1.2337	1.7043	1.7032	1.7100	1.2271
1.0	1.2481	1.7345	1.7363	1.7635	1.2387
			*m*_2_ = 3.5		
0.5	1.2633	2.0334	2.0254	2.0485	1.2569
1.0	1.2777	2.0810	2.0634	2.1225	1.2668
			*m*_2_ = 4.0		
0.5	1.2925	2.4705	2.4250	2.4753	1.2861
			*AAD%* = 0.78	*AAD%* = 1.04	*AAD%* = 0*.*34
			*SD* = 0.0127	*SD* = 0.0154	*SD* = 0*.*0047

The standard uncertainties (*u*) are *u*(*T*) = 0.02 K, *u(m)* = 1.0 × 10^−4^ mol kg^−1^. The combined expanded uncertainty (*U*_*c*_) are *U*_*c*_(*ρ*) = 5.0 × 10^−5^ g cm^−3^ (0.95 level of confidence) and U_c_($\eta$) = 0.003 mPa s (0.95 level of confidence).

**Table 8:** Experimental and calculated densities $\rho$ and Viscosities $\eta$ at T = 318.15 K for KCl (1) + CaCl_2_ (2) + H_2_O (3) SYSTEM.

*m*_1_ (mol kg^−1^)	Experimental data		Calculated viscosity (mPa s)		Predicted density
	$\rho$ (g cm^−3^)	$\eta$ (mPa s)	Equation (5)	Hu’s model	$\rho$ (g cm^−3^)
			*m*_2_ = 0*.*5		
0.5	1.0539	0.7214	0.7087	0.7140	1.0566
1.0	1.0735	0.7345	0.7207	0.7272	1.0764
1.5	1.0923	0.7434	0.7331	0.7408	1.0950
2.0	1.1101	0.7537	0.7458	0.7547	1.1128
2.5	1.1272	0.7656	0.7591	0.7691	1.1299
3.0	1.1435	0.7851	0.7739	0.7846	1.1465
3.5	1.1588	0.8057	0.7887	0.7994	1.1626
			*m*_2_ = 1.0		
0.5	1.0925	0.8341	0.8258	0.8474	1.0955
1.0	1.1105	0.8404	0.8405	0.8612	1.1133
1.5	1.1276	0.8566	0.8552	0.8751	1.1305
2.0	1.1441	0.8865	0.8720	0.8911	1.1477
2.5	1.1592	0.8970	0.8866	0.9045	1.1639
3.0	1.1737	0.9077	0.9035	0.9197	1.1802
			*m*_2_ = 1.5		
0.5	1.1283	0.9716	0.9647	0.9895	1.1307
1.0	1.1447	0.9840	0.9822	1.0043	1.1480
1.5	1.1600	0.9917	0.9993	1.0190	1.1649
2.0	1.1746	1.0251	1.0176	1.0347	1.1818
2.5	1.1883	1.0496	1.0363	1.0504	1.1987
			*m*_2_ = 2.0		
0.5	1.1619	1.1300	1.1319	1.1481	1.1653
1.0	1.1765	1.1653	1.1518	1.1649	1.1827
1.5	1.1902	1.1680	1.1717	1.1818	1.2000
2.0	1.2032	1.1964	1.1945	1.2009	1.2180
			*m*_2_ = 2.5		
0.5	1.1935	1.3299	1.3340	1.3374	1.1998
1.0	1.2064	1.3538	1.3563	1.3571	1.2180
1.5	1.2183	1.3826	1.3792	1.3767	1.2365
			*m*_2_ = 3.0		
0.5	1.2237	1.5666	1.5818	1.5763	1.2344
1.0	1.2347	1.6321	1.6063	1.5984	1.2538
			*m*_2_ = 3.5		
0.5	1.2528	1.8659	1.8879	1.8827	1.2682
1.0	1.2619	1.9129	1.9156	1.9053	1.2894
			*m*_2_ = 4.0		
0.5	1.2812	2.2479	2.2726	2.2750	1.3005
			*AAD%* = 0.90	*AAD%* = 0.99	*AAD%* = 0.39
			*SD* = 0.0130	*SD* = 0.0138	*SD* = 0.0054

The standard uncertainties (*u*) are *u*(*T*) = 0.02 K, *u(m)* = 1.0 × 10^−4^ mol kg^−1^. The combined expanded uncertainty (*U*_*c*_) are *U*_*c*_(*ρ*) = 5.0 × 10^−5^ g cm^−3^ (0.95 level of confidence) and U_c_($\eta$) = 0.003 mPa s (0.95 level of confidence).

**Table 9:** Experimental and calculated densities $\rho$ and viscosities $\eta$ at T = 323.15 K for KCl (1) + CaCl_2_ (2) + H_2_O (3) system.

*m*_1_ (mol kg^−1^)	Experimental data		Calculated viscosity (mPa s)		Predicted density
	$\rho$ (g cm^−3^)	$\eta$ (mPa s)	Equation (5)	Hu’s model	$\rho$ (g cm^−3^)
			*m*_2_ = 0*.*5		
0.5	1.0507	0.6662	0.6551	0.6633	1.0542
1.0	1.0714	0.6771	0.6689	0.6754	1.0756
1.5	1.0902	0.6892	0.6828	0.6881	1.0956
2.0	1.1083	0.7004	0.6970	0.7010	1.1146
2.5	1.1297	0.7134	0.7116	0.7140	1.1324
3.0	1.1424	0.7284	0.7274	0.7275	1.1494
4.0	1.1583	0.7544	0.7430	0.7396	1.1653
			*m*_2_ = 1.0		
0.5	1.0907	0.7685	0.7648	0.7836	1.0917
1.0	1.1088	0.7825	0.7819	0.7968	1.1110
1.5	1.1273	0.7950	0.7989	0.8097	1.1290
2.0	1.1436	0.8099	0.8178	0.8238	1.1464
2.5	1.1601	0.8272	0.8344	0.8347	1.1622
3.0	1.1756	0.8434	0.8530	0.8462	1.1773
			*m*_2_ = 1.5		
0.5	1.1271	0.8906	0.8931	0.9176	1.1271
1.0	1.1445	0.9092	0.9138	0.9310	1.1444
1.5	1.1613	0.9217	0.9338	0.9430	1.1605
2.0	1.1772	0.9518	0.9550	0.9546	1.1758
2.5	1.1926	0.9603	0.9764	0.9646	1.1900
			*m*_2_ = 2.0		
0.5	1.1416	1.0246	1.0452	1.0715	1.1604
1.0	1.1788	1.0783	1.0690	1.0835	1.1758
1.5	1.1943	1.0826	1.0927	1.0934	1.1900
2.0	1.2095	1.1095	1.1188	1.1032	1.2035
			*m*_2_ = 2.5		
0.5	1.1968	1.2266	1.2261	1.2531	1.1920
1.0	1.2117	1.2549	1.2530	1.2611	1.2054
1.5	1.2267	1.2846	1.2803	1.2657	1.2178
			*m*_2_ = 3.0		
0.5	1.2283	1.4415	1.4437	1.4718	1.2223
1.0	1.2427	1.4802	1.4735	1.4699	1.2337
			*m*_2_ = 3.5		
0.5	1.2578	1.7161	1.7070	1.7327	1.2516
1.0	1.2720	1.7557	1.7407	1.7092	1.2609
			*m*_2_ = 4.0		
0.5	1.2868	2.0785	2.0306	2.0326	1.2802
			*AAD%* = 0.79	*AAD%* = 1.31	*AAD%* = 0.40
			*SD* = 0.0124	*SD* = 0.0199	*SD* = 0.0056

The standard uncertainties (*u*) are *u*(*T*) = 0.02 K, *u(m)* = 1.0 × 10^−4^ mol kg^−1^. The combined expanded uncertainty (*U*_*c*_) are *U*_*c*_(*ρ*) = 5.0 × 10^−5^ g cm^−3^ (0.95 level of confidence) and U_c_($\eta$) = 0.003 mPa s (0.95 level of confidence).

**Figure 1:**
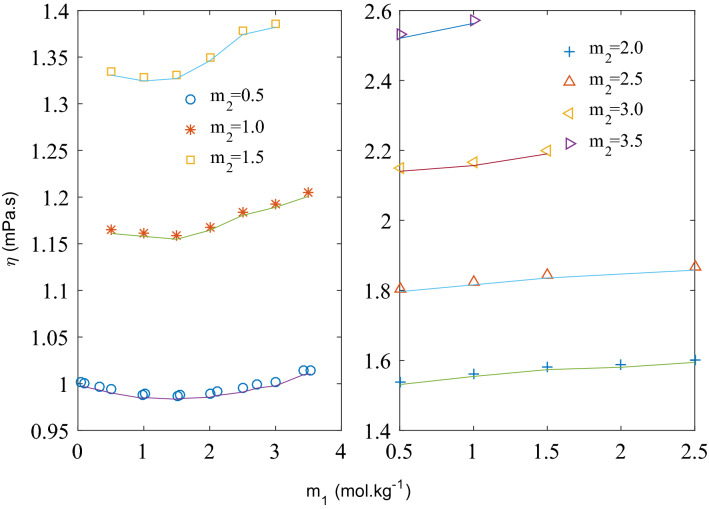
Viscosity of ternary aqueous solution of KCl (*m*_1_) and CaCl_2_ (*m*_2_) at 293.15 K. Solid line to show trend only.

In Fig. 1, viscosity of ternary solution is plotted at fixed molalities of CaCl_2_. At low concentration of CaCl_2_ (m_2_ = 0.5–1.0 mol kg^−1^), viscosity of the ternary solution decreases initially with increasing molality of KCl reaching a minimum value, and then starts increasing monotonically. Viscosity data taken from literature^20^ of binary solution of KCl (m_2_ = 0 mol kg^−1^) is also shown on the same figure . As it can be observed that viscosity of the binary solution also decreases initially to a minimum at around m_1_ = 1.5 mol kg^−1^ and then starts increasing. This trend is attributed to structure breaking property of K^+^ (negative value of Jones Dole B coefficient) in binary solution reported by many researchers^20–25^. At lower concentration (up to 1.0 mol kg^−1^) of CaCl_2_, addition of KCl affects the viscosity in a similar manner as the binary KCl solution. Hence, it can be assumed that structure of the ternary solution at low concentration of CaCl_2_ is close to that of the binary KCl solution. It is very clear from Fig. 1 that extent of structure breaking property of KCl is diminishing; lower negative slope in decreasing region and higher positive slope in increasing region of viscosity as concentration of CaCl_2_ increasing from zero to 1.0 mol kg^−1^. Figure 1 shows a presentation of the trend of viscosity decrease and increase to clarify the idea and not finding the slope for the data points. Moving to higher concentration of CaCl_2_, initial decrease in viscosity is not observed with increase in KCl molality as at higher concentration solute–solute interaction is supposed to dominate. Viscosity of ternary solution increases with increase in KCl concentration for CaCl_2_ concentration (m_2_) more than 1.5 mol kg^−1^ as shown in Fig. 1.

**Figure 2:**
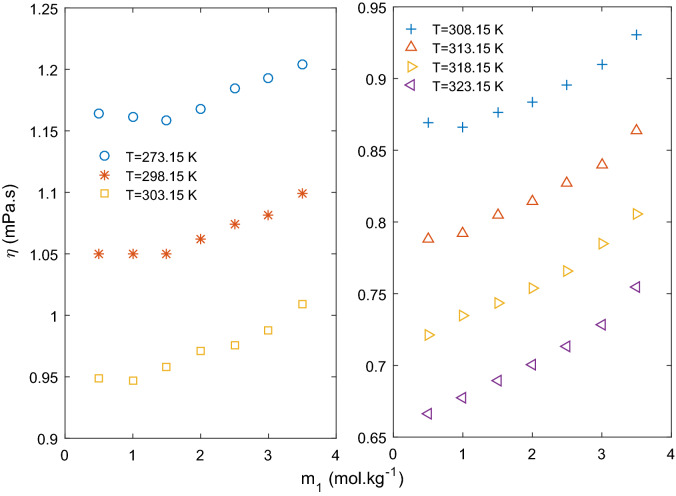
Viscosity of ternary aqueous solution of KCl (*m*_1_) and constant CaCl_2_ (*m*_2_) = 0.5 mol kg^−1^ at different temperatures*.*

Variation of viscosity with KCl concentration (m_1_) at different temperatures is presented in Fig. 2. It can be seen that a minima is observed at lower temperatures up to 308.15 K but after this temperature viscosity increased as concentration of KCl increases. At higher temperature, structure-breaking property of K^+^ reduces as water structure is destroyed. Jones–Dole coefficient B values for KCl is found to be negative at lower temperatures and positive at higher temperatures as presented in Table 10. Temperature dependent structure breaking property of KCl and other similar electrolyte has been reported in literature and discussed in details^18^.

**Table 10:** Coefficients (A–D) of the extended Jones Dole Eq. (4) of KCl + H_2_O Data at different temperatures.

T/K	A (mol^−1/2^ kg^1/2^)	B (mol^−1^ kg^−1^)	10^3^D (mol^−2^ kg^2^)	AAD (%)
293.15	0.00498	− 0.028	8.06 ± 0.20	0.27
298.15	0.0051	− 0.014	6.27 ± 0.07	0.15
303.15	0.00514	− 0.002	4.87 ± 0.14	0.21
308.15	0.0052	0.008	3.48 ± 0.19	0.16
313.15	0.0053	0.017	2.95 ± 0.16	0.29
318.15	0.00539	0.026	2.28 ± 0.26	0.40
323.15	0.00547	0.033	1.58 ± 0.20	0.24

### Modeling

In the previous work^18^, viscosity data of the ternary solutions of NaCl and CaCl_2_ were correlated by three models available in the literature; the mixing model (GF model) developed by Goldsack and Franchetto^5,26^, the exponential model and the extended Jones–Dole model^27^. The present work, viscosity data of the ternary aqueous solution of potassium chloride and calcium chloride are correlated and predicted by the above mentioned models in addition to Hue equation^28^ and modified extended Jones Dole.

Four models are presented in this work; two are purely predictive (GF model and Hue model). They use data of corresponding binary system and then predict the viscosity of ternary solution. While the exponential model is purely empirical as model coefficients are calculated by fitting the experimental data. The fourth model proposed in Eq. (5) can be called semi empirical as it used A–F values of binary system but G value is calculated by correlating with experimental data.

MATLAB non-linear fit tool NLINFIT was used to find the model coefficient of all models reported in this work. NLINFIT function least squares to estimate the coefficients of a nonlinear regression function. Viscosity is the dependent variable while m_1_ and m_2_ are the independent variables. Initial guesses of the coefficients as input are required. NLINFIT returns the predicted values of the viscosity along with the estimated coefficients. NLPREDCI and NLPARCI tools in MATLAB are used to calculate the confidence intervals for the predicted values.

#### Modified extended Jones Dole model

Zhangh et al.^19^ extended the Jones Dole equation (Eq. 2) to be applicable for ternary solutions. The method calculates the viscosity of ternary solution by simple additive rules as shown in Eq. (3).

3 $$ \frac{\eta }{{\eta_{0} }} = 1 + A_{1} m_{1}^{1/2} + B_{1} m_{1} + D_{1} m_{1}^{2} + E_{1} m_{1}^{3.5} + F_{1} m_{1}^{7} + A_{2} m_{2}^{1/2} + B_{2} m_{2} + D_{2} m_{2}^{2} + E_{2} m_{2}^{3.5} + F_{2} m_{2}^{7} $$

where *m* is the concentration in mol kg^−1^, subscript 1 and 2 are for the corresponding binary solutions. *A and B* values were taken from^22,29^ and the other coefficients of the model are calculated by fitting binary data to the corresponding equation for binary mixture as below.

4 $$ \frac{\eta }{{\eta_{0} }} = 1 + Am^{1/2} + Bm + Dm^{2} + Em^{3.5} + Fm^{7} $$

In the previous work^18^, it has been shown that Eq. (3) failed to predict the corresponding viscosity of ternary solutions especially at higher concentrations. Calculated viscosity by Eq. (3) was found to be much smaller than the experimental viscosity of the ternary solution and at higher concentration deviation goes up to − 15% for the ternary system of CaCl_2_ and NaCl. The discrepancy was due to the ions interaction at higher concentrations. In dilute solutions, enough water is available to hydrate all ions but in concentrated ones, the interaction of ions becomes stronger and hence real concentration of one electrolyte should be more due to the competition of the electrolyte ions for the available water molecules. Therefore, the reason behind the discrepancy of viscosity predictions using Eq. (3) could be due to the large differences between hydrating abilities of Ca^2+^ and Na^+^/K^+^, strong repulsion between Ca^2+^ and Na^+^/K^+^ and greater difference of size and shape of Ca^2+^ and Na^+^/K^+^ ions^19^. So in order to minimize the deviations, an extra term (G) is introduced in Eq. (3), which can be called ions interaction parameter. Similar models with interaction coefficient are reported in literature for viscosity of liquids mixtures and Interaction coefficient is found to be temperature dependent only^30^. The modified equation can be expressed as below

5 $$ \eta = \eta_{0} \left( {1 + A_{1} m_{1}^{\frac{1}{2}} + B_{1} m_{1} + D_{1} m_{1}^{2} + E_{1} m_{1}^{3.5} + F_{1} m_{1}^{7} + A_{2} m_{1}^{\frac{1}{2}} + B_{2} m_{1} + D_{2} m_{1}^{2} + E_{2} m_{1}^{3.5} + F_{2} m_{1}^{7} } \right) + Gm_{1} m_{2} $$

Application of the calculation method proposed in Eq. (5) can be summarized in the following steps.

1. Getting A–F values in Eq. (4) for binary solutions: Jones–Dole coefficients A and B values for a particular binary solution can be obtained from literature to be used in Eq. (5). Using these values of A and B, viscosity data of binary solution can be regressed to Eq. (4) to obtain D–F values.
2. Getting term G: Once values of A–F are obtained for both binary solutions, these values are inserted in Eq. (5) and thus only the term G remains unknown. G can be obtained by regressing the viscosity data of ternary solutions against Eq. (5).

The above-mentioned methodology was applied to the measured data of ternary aqueous solution of KCl + CaCl_2_. KCl binary viscosity were taken from collection of data by Laliberté^31^. The values of coefficients A–D in Eq. (4) was good enough to correlate experimental viscosity data. Zhangh et al.^19^ used Eq. (4) to correlate the viscosity of KCl binary solution, but after investigation, it was found that dropping the terms E and F affected the AAD % only by 0.1%. Therefore, the last two terms in Eq. (4) were dropped from the viscosity calculation. The coefficients were presented in Table 10 and AAD% of binary experimental viscosity data correlated to Eq. (4) was included in Table 10, too. Equation (6) was used to calculate the average absolute deviation (*AAD*);

6 $$ AAD = \left[ {\sum {\frac{{\left| {\eta_{\exp ,i} - \eta_{cal,i} } \right|}}{{\eta_{\exp ,i} }}} } \right]\frac{100}{n} $$

where n is the number of data points.

For CaCl_2_ binary data taken from^32^, coefficients A–F in Eq. (4) were required to correlate viscosity data accurately. The coefficients are presented in the Table 11 along with the AAD% of binary experimental viscosity data correlated to Eq. (4). The AAD% in both Tables 10 and 11 are less than 0.5%.

**Table 11:** Coefficients (A–F) of the extended Jones Dole Eq. (4) of CaCl_2_ + H_2_O Data at different temperatures.

T/K	A (mol^−1/2^ kg^1/2^)	B (mol^−1^ kg^−1^)	D (mol^−2^ kg^2^)	10^3^ E (mol^−3.5^ kg^3.5^)	10^6^ F (mol^−7^ kg^7^)	AAD (%)
293.15	0.01511	0.236	0.056 ± 0.003	4.68 ± 0.60	7.61 ± 1.12	0.34
298.15	0.0155	0.261	0.046 ± 0.003	5.48 ± 0.51	5.93 ± 0.95	0.25
303.15	0.0156	0.256	0.056 ± 0.004	4.59 ± 0.73	6.15 ± 1.38	0.28
308.15	0.0158	0.265	0.054 ± 0.008	4.61 ± 0.18	5.44 ± 1.16	0.43
313.15	0.0161	0.274	0.053 ± 0.003	4.62 ± 0.49	4.74 ± 0.92	0.28
318.15	0.01633	0.282	0.054 ± 0.004	4.3 ± 0.71	4.89 ± 1.33	0.36
323.15	0.01658	0.289	0.054 ± 0.007	3.89 ± 0.15	5.03 ± − 2.16	0.31

**Figure 3:**
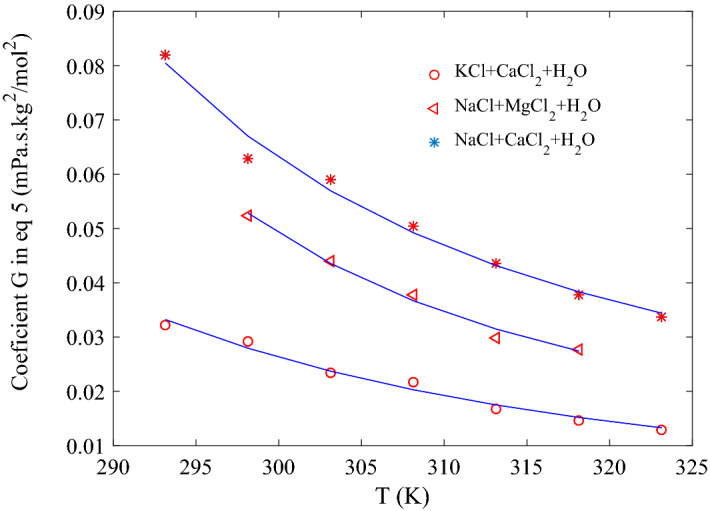
Variation of term G in Eq. (5) for the ternary systems KCl + CaCl_2_ + H_2_O, NaCl + MgCl_2_ + H_2_O and NaCl + CaCl_2_ + H_2_O against temperature.

Calculated values of A–F for the two binary systems were inserted in Eq. (5), which was regressed against measured viscosity values of ternary system using least square method. Optimized values of parameter G were obtained and presented in Table 12 and Fig. 3. Values of G was found to be decreasing with increase in temperature which means that interaction of ions is weakening at higher temperature.

**Table 12:** Coefficient G in Eq. (5) as a function of temperature for different ternary systems.

T (K)	KCl + CaCl_2_	(NaCl + CaCl_2_)		NaCl + MgCl_2_	
	G (mPa s mol^−2^ kg^2^)	G (mPa s mol^−2^ kg^2^)	AAD (%)	G (mPa s mol^−2^ kg^2^)	AAD (%)
293.15	0.0322 ± 0.0045	0.0819 ± 0.0061	1.63	–	–
298.15	0.0293 ± 0.0045	0.0629 ± 0.0057	1.69	0.0524 ± 0.0032	0.92
303.15	0.0234 ± 0.0034	0.0591 ± 0.0044	1.45	0.044 ± 0.0028	0.96
308.15	0.0217 ± 0.0029	0.05043 ± 0.0036	1.44	0.0378 + 0.0024	0.81
313.15	0.0167 ± 0.0022	0.0436 ± 0.0033	1.33	0.0298 + 0.0067	1.63
318.15	0.0146 ± 0.0018	0.0378 ± 0.0032	1.32	0.0278 + 0.002	0.87
323.15	0.0130 ± 0.0021	0.0337 ± 0.00259	1.25	–	–

Viscosity of ternary system of KCl + CaCl_2_ + H_2_O calculated by Eq. (5) to be found in column 4 in all Tables 3, 4, 5, 6, 7, 8 and 9. Experimental and calculated data were compared and the standard deviation (*SD*) was calculated using Eqs. (7).

7 $$ SD = \left[ {\frac{{\sum\limits_{i = 1}^{n} {\left( {\eta_{\exp ,i} - \eta_{cal,i} } \right)^{2} } }}{n - p}} \right]^{1/2} $$

**Figure 4:**
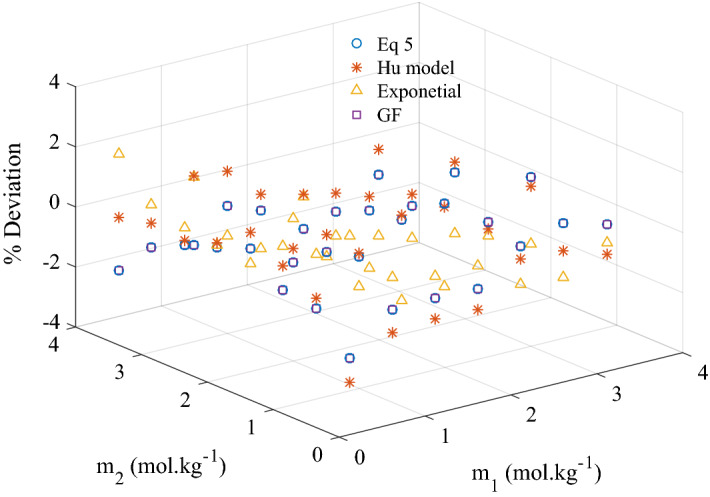
Percentage deviation for the calculated viscosity of ternary system of KCl + CaCl_2_ + H_2_O by different models for experimental data at 303.15 K.

**Figure 5:**
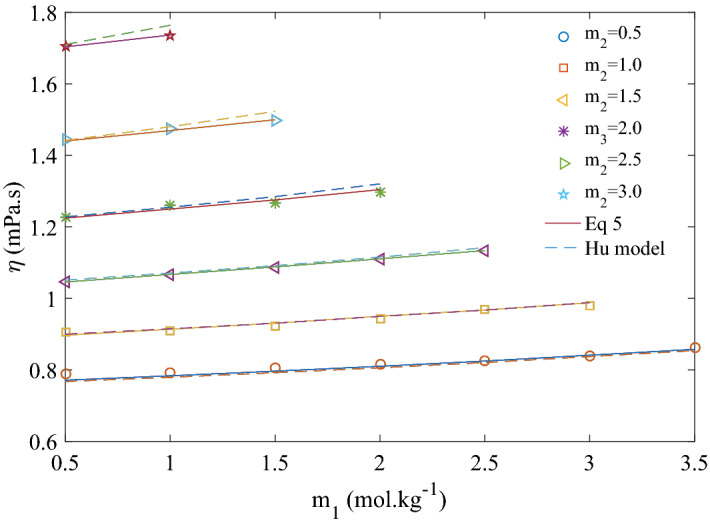
Experimental data and correlated values of viscosity of ternary aqueous solution of KCl (*m*_1_) and CaCl_2_ (*m*_2_) at 313.15 K. Solid lines are for Eq. (5) and dotted line are Hu model, Eq. (8).

*p* represents the number of adjusted parameters. Values of *SD* and *AAD* are reported at the bottom of Tables 3, 4, 5, 6, 7, 8 and 9. *SD* values were found to be varying from 0.0124 to 0.0255. AAD% was found to be less than 1.0% with maximum deviation of around 2.0% at high concentrations of both solutes as shown in Fig. 4. In Fig. 5, experimental data of viscosity and viscosity predicted by Eq. (5) were presented. Analyzing statistical data along with Figs. 4 and 5 it can be concluded that with the introduction of interaction parameter G in Eq. (5) is predicting the viscosity data of ternary solution very well compared to Eq. (3).

The proposed model (Eq. 5) was also validated against another well-known binary system; NaCl + CaCl_2_ + H_2_O based on the published data of the ternary system^18^. A–E coefficient for the binary NaCl solution were reported in Table 13. A–B values were taken from Aleksandrov et al.^33^ and D–E coefficient values were obtained by regression of Eq. (4) to the viscosity data taken from Kestin et al.^34^. After fitting viscosity data of ternary NaCl + CaCl_2_ + H_2_O the value of the empirical parameters in Eq. (5) was obtained. Values of G along with Absolute average deviation (AAD) between experimental and predicted values by Eq. (5) were also presented in Table 13 and Fig. 3. It can be concluded that Eq. (5) successfully predicted the viscosity of ternary solution of NaCl + CaCl_2_ + H_2_O as AAD were less than 2% for all temperatures. G values for the ternary system NaCl + CaCl_2_ + H_2_O were found to be higher than that of KCl + CaCl_2_ + H_2_O suggesting stronger interaction for the former compared to the later.

**Table 13:** Coefficients (A–E) of extended Jones Dole equation (Eq. 4) for NaCl + H_2_O Data at different temperatures.

T/K	A (mol^−1/2^ kg^1/2^)	B (mol^−1^ kg^−1^)	10^3^D (mol^−2^ kg^2^)	10^5^E (mol^−3.5^ kg^3.5^)	AAD (%)
293.15	0.0074	0.063	14.5 ± 0.34	7.50 ± 2.91	0.12
298.15	0.0075	0.075	11.4 ± 0.21	16.0 ± 1.73	0.14
303.15	0.0071	0.081	10.6 ± 0.32	14.0 ± 2.58	0.11
308.15	0.0068	0.088	9.23 ± 0.39	18.20 ± 4.04	0.07
313.15	0.0066	0.09	9.47 ± 2.86	10.70 ± 2.86	0.10
318.15	0.0057	0.095	9.31 ± 0.27	9.41 ± 2.77	0.05
323.15	0.005	0.1	6.47 ± 0.38	25.6 ± 4.19	0.09

**Figure 6:**
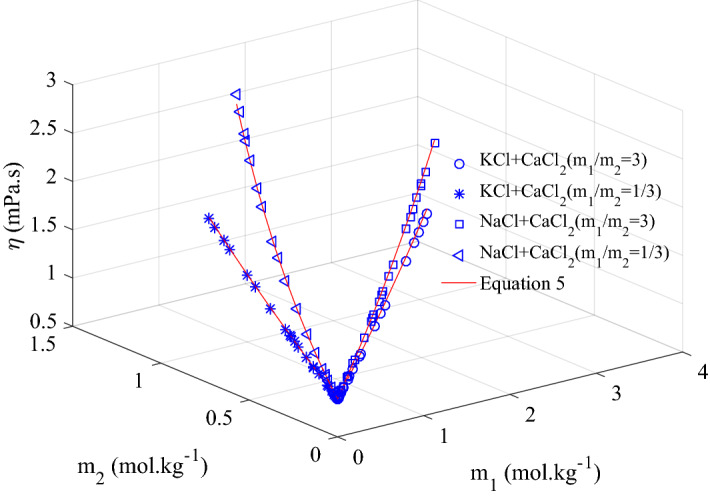
Comparison of calculated viscosity using Eq. (5) for ternary system of KCl + CaCl_2_ + H_2_O and published viscosity data^19^ at 298.15 K.

Calculation method proposed in Eq. (5) was validated against published data^27^. Viscosity of ternary mixtures KCl + CaCl_2_ + H_2_O and NaCl + CaCl_2_ + H_2_O was calculated by Eq. (5) at the concentrations reported in the published data^27^ by using A–F and G values presented in Tables 11, 12 and 13 and compared with the measured reported data. Calculated and published measured viscosity are found to be very close with AAD equal to 0.85% for KCl + CaCl_2_ + H_2_O system and 1.17% for NaCl + CaCl_2_ + H_2_O. Maximum deviation of 3.9% and 3.2% was found for the systems KCl + CaCl_2_ + H_2_O and NaCl + CaCl_2_ + H_2_O respectively. This comparison is presented in Fig. 6, which confirms that Eq. (5) is highly accurate in predicting the viscosity data already presented in the literature.

Equation (5) was also successfully applied to regress ternary viscosity mixture NaCl + MgCl_2_ published data^3^. Ternary data for this system are only available in the range of temperature 298.15–318.15 K and hence G values for these temperatures are reported in Table 12 along with AAD for experimental data and values obtained by Eq. (5).

So, the overall method proposed in Eq. (5) was validated for viscosity of three ternary systems; KCl + CaCl_2_ + H_2_O, NaCl + CaCl_2_ + H_2_O, and NaCl + CaCl_2_ + H_2_O. G Coefficient, which is a measure of the overall deviation arising in additive methods of calculating viscosity of ternary solution (Eq. 3), is plotted against temperature in Fig. 3. Ion interaction Coefficient G is found to be decreasing with temperature for all systems. To investigate further the G dependency on temperature, the following model is used to correlate it with temperature.

8 $$ G = Ae^{{\frac{B}{{\left( {T - C} \right)}}}} $$

Optimized value of model coefficients of Eq. (8) are found to be; A = 5.04E−05, B = 1182.78, C = 110.90 for KCl + CaCl_2_ + H2O, A = 0.00208, B = 254.06, C = 219.54 for NaCl + CaCl_2_ + H_2_O and A = 0.003571, B = 249.02, C = 213.20. Therefore, it is clear that temperature dependence of G coefficients for all three systems follows the same trend (see Fig. 3). At a fixed temperature, different values of G for different ternary system suggest that it is governed by properties of cations present in that system. These properties of cations include the cation hydration extent, difference of shape and size, and repulsive forces. More ternary system should be investigated to find out which of the above factor(s) is/are significant in controlling the ion coefficient parameter.

#### Hu equation

Hu and Lee^35^ has proposed simple predictive equation for viscosity of mixed electrolyte solution based on the absolute rate theory^36^ and the equation of Patwardhan and Kumar^37^. Hu equation can be expressed as

9 $$ \ln \eta = \mathop \sum \limits_{i = 1}^{i = j} \frac{{x_{i} }}{{x_{i}^{o} }}\ln \eta_{i}^{o} $$

where $\eta_{i}^{o}$ is the viscosity of $i$ binary solution having the same ionic strength as that of mixed solution, $x_{i}^{o}$ is the mole fraction of $i$ in binary solution($i$-H_2_O) having the same ionic strength as that of mixed solution, $x_{i}$ is the mole fraction of $i$ in the binary solution ($i$-H_2_O).

Measured values of ternary aqueous solution viscosity of KCl + CaCl_2_ were used to test Eq. (9). The procedure is briefly summarized as follows ^38^:

1. Fit the available data^19,32^ of measured viscosity for binary solution by the following equations:
  10 $$ \eta_{i}^{o} = \sum\limits_{i = 0}^{i = k} {A_{i} } \left( {m_{i}^{o} } \right)^{i/2} $$
  where $\eta_{i}^{o}$ and $m_{i}^{o}$ represent the viscosity and the molality of the binary aqueous solution. The optimum fit was obtained by varying $A_{i}$ s until the values of $\partial_{\eta ,i}$ is less than 10–4. $\partial_{\eta ,i}$ is calculated according to Eq. (11).
  11 $$ \delta_{\eta ,i} = \frac{{\frac{{\mathop \sum \nolimits_{j = 1}^{n} \left( {\left| {\eta_{{i\left( {cal} \right)}}^{o} - \eta_{{i\left( {exp} \right)}}^{o} } \right|} \right)}}{{\eta_{{i\left( {exp} \right)}}^{o} }}}}{n} $$
  The values of $A_{i}$ obtained for binary solutions are shown in the Table 14.
2. Determine the composition ${{m}_{i}}^{o}$ of the binary solutions with the same ionic strength as that of ternary solution of given molalities $m_{i} (i = 1,2).$
3. Compare predicted and measured data.

**Table 14:** Coefficients of A_i_’s in Eq. (10) for the binary solutions of KCl + H_2_O and CaCl_2_ + H_2_O.

T/ K	293.15	298.15	303.15	308.15	313.15	318.15	323.15
**KCl + H**_**2**_**O**							
A_0_ (mPa s)	1.001	0.891	0.798	0.720	0.661	0.653	0.540
10^2^A_1_ (mPa s kg^1/2^ mol^−1/2^)	1.31	− 0.092	− 0.062	0.117	− 0.177	− 0.141	2.25
10^4^A_2_ (mPa s kg^1^ mol^−1^)	− 490	0.988	1.01	0.83	0.78	0.79	1.04
10^2^A_3_ (mPa s kg^3/2^ mol^−3/2^)	2.48	− 2.00	1.17	1.86	− 1.72	2.24	− 0.18
10^3^A_4_ (mPa s kg^2^ mol^−2^)	− 1.09	20.7	− 5.75	− 23.7	44.0	− 10.2	8.00
10^3^A_5_ (mPa s kg^5/2^ mol^−5/2^)	0.49	− 3.82	1.84	15.80	2.35	2.07	− 1.50
10^5^A_6_ (mPa s kg 3 mol^−3^)	7.00	− 0.0063	4.980	− 324.00	415	4.48	− 16.3
**CaCl**_**2**_ **+ H**_**2**_**O**							
A_0_ (mPa s)	1.207	1.085	0.9745	0.718	0.742	0.535	0.553
10^2^A_1_ (mPa s kg^1/2^ mol^−1/2^)	− 0.219	− 0.387	0.0797	0.0635	− 0.1268	0.2683	0.0297
10^4^A_2_ (mPa s kg^1^ mol^−1^)	− 0.859	− 0.213	0.0002	− 0.0253	− 0.198	0.0735	0.0037
10^2^A_3_ (mPa s kg^3/2^ mol^−3/2^)	2.345	1.541	0.0065	0.430	0.966	− 0.908	0.3205
10^3^A_4_ (mPa s kg^2^ mol^−2^)	− 1.471	− 1.1162	0.0404	− 0.3143	− 0.666	1.603	− 0.171
10^3^A_5_ (mPa s kg^5/2^ mol^−5/2^)	0.289	0.264	− 0.0268	0.1034	0.1612	− 0.948	0.0175
10^5^A_6_ (mPa s kg 3 mol^−3^)	0.0280	0.0132	0.0047	0.0039	0.0072	0.2004	0.0167

Comparison between experimental viscosity and predicted one were made by calculating average absolute deviation (AAD%) and standard deviation (SD) and reported in Tables 3, 4, 5, 6, 7, 8 and 9. Maximum AAD and SD was found to be 1.31% and 0.0199 m Pa s respectively. Experimental data and calculated viscosity were plotted in Fig. 5. The deviations in Fig. 4 suggest that Hue equation predicts the viscosity of ternary solution reasonably accurate.

#### Exponential model

Many researcher^27,39–41^ have used the following semi-empirical exponential model and successfully correlated the model with the viscosity of sodium and calcium solution and sodium and magnesium solution at high concentrations of salts^18^.

12 $$ \eta = aexp\;(b_{1} m_{1} + f_{1} m_{1}^{2} + b_{2} m_{2} + f_{1} m_{2}^{2} ) $$

where *a*, *b*_1_, *b*_2_, *f*_1_ and *f*_2_ are the model coefficients. In this work, Eq. (12) was correlated to the data presented in Tables 3, 4, 5, 6, 7, 8 and 9 at all studied temperatures.

The coefficients; *b*_1_, *b*_2_, *f*_1_ and *f*_2_ in Eq. (12) were found to be temperature independent and have constant values, but the coefficient *a* was found to be temperature dependent. The optimized values of *b*_1_, *f*_1_*, b*_2_ *and f*_2_ are 0.0302 ± 0.0055, − 0.0005 ± 0.0019, 0.2726 ± 0.0040 and 0.0132 ± 0.0018, respectively. Temperature dependence of parameter *a* in Eq. (12) was modeled by Eq. (13). Coefficient *a* values at different temperatures are presented in Table 15.

13 $$ a = a_{0} \exp \left( {\frac{{a_{1} }}{{T - a_{2} }}} \right) $$

**Table 15:** Values of the coefficient *a* (Eq. 13) at the studied temperatures for KCl + CaCl_2_ + H_2_O.

T(K)	a (mPa s)
293.15	0.9788 ± 0.0056
298.15	0.8835 ± 0.0034
303.15	0.7979 ± 0.0024
308.15	0.7275 ± 0.0019
313.15	0.6648 ± 0.0020
318.15	0.6123 ± 0.0032
323.15	0.5657 ± 0.0034

where *a*_0_, *a*_1_ and *a*_2_ are adjustable parameters. The calculated values for the ternary KCl + CaCl_2_ + H_2_O are 0.0302 ± 0.012, 557.1013 ± 138.7 and 132.9 ± 21.5 for *a*_0_, *a*_1_ and *a*_2_ respectively. Consequently, the final temperature dependent viscosity model can be expressed as follows.

14 $$ \eta = a_{0} \exp \;\left( {\frac{{a_{1} }}{{T - a_{2} }}} \right)\exp {\kern 1pt} \;\left( {b_{1} m_{1} + f_{1} m_{1}^{2} + b_{2} m_{2} + f_{2} m_{2}^{2} } \right) $$

Viscosity of the ternary solution calculated by Eq. (14) was compared with the measured viscosity of KCl + CaCl_2_ + H_2_O in this work. The maximum value of AAD was found to be 1.63% at the temperature of 293.15 K, otherwise it is less. Percentage deviation plot as depicted in Fig. 4 against all the concentrations at the temperature of 303.15 K (as an example) suggest that maximum deviation was 1.87%. These statistical data above leads to the conclusion that Eq. (13) fits well the experimental data.

#### Goldsack and Frachetto model (GF model).

Many researcher has adapted a model based on absolute rate theory derived by Goldsack and Frachetto^23^ to predict the viscosity of electrolyte mixtures^42,43^. The equation is as follows:

15 $$ \eta = \frac{{\eta_{w} e^{{\left( {X_{1} E_{1} + X_{2} E_{2} } \right)}} }}{{(1 + X_{1} V_{1} + X_{2} V_{2} )}} $$

*X*_1_ and *X*_2_ are the mole fractions presented in the following two equations for a solution made of two electrolyte 1 and 2, while *E* and *V* are dimensionless free energy and volume parameters, subscript 1 and 2 are for the corresponding binary solutions.

16 $$ X_{1} = \frac{{m_{1} }}{{55.51 + \nu {}_{1}m_{1} + \nu {}_{2}m_{2} }} $$

17 $$ X_{2} = \frac{{m_{2} }}{{55.51 + \nu {}_{1}m_{1} + \nu {}_{2}m_{2} }} $$

Values of $\nu {}_{1}$ and $\nu_{2}$ are 2 and 3 respectively while E and V parameters of corresponding binary solution can be calculated by regressing the binary viscosity data against corresponding Goldsack and Frachetto viscosity model for binary solution as expressed in Eq. (18):

18 $$ \eta = \frac{{\eta_{w} e^{XE} }}{1 + XV} $$

Electrolytes with a total of two ions (1:1 electrolyte) like NaCl, KCl and MgSO_4_ , mole fraction of cations is calculated as:

19 $$ X = \frac{m}{55.51 + 2m} $$

Electrolytes with a total of three ions (1:2 electrolyte) like CaCl_2_, MgCl_2_ and Ca(NO_3_)_2_, mole fraction of cations can be calculated as:

20 $$ X = \frac{m}{55.51 + 3m} $$

Equations (15) to (20) were used to predict the viscosity of the ternary solutions of potassium and calcium chlorides.

Regressing the binary data for KCl^23^ to Eqs. (18) and (19), *E* and *V* values were calculated and reported in Table 16. *E*_2_ and *V*_2_ values that correspond for CaCl_2_ binary data were taken from the work^18^. The calculated values were inserted into Eqs. (14) to (16) in order to predict the viscosity of ternary mixtures. Validity of GF model against KCl + CaCl_2_ + H_2_O viscosity data was investigated by calculating AAD. Maximum AAD was found to be 2.3% at 318.15 K. A representative plot for percentage deviation between the measured and the calculated viscosities was shown in Fig. 4. It suggests that at two points, deviation crosses 3.0% (3.7% and 3.3%) otherwise it is around 1.0% at other points.

**Table 16:** Values of *E* and *V* at different temperatures for KCl + CaCl_2_ + H_2_O.

*T*/K	*E*_1_	*V*_1_	*E*_2_	*V*_2_	*E*_2_	*V*_2_
			Molality 0–2	Molality 0–2	Molality 2–5	Molality 2–5
293.15	7.60	9.12	28.44	12.00	43.44	42.98
298.15	7.69	8.54	27.04	9.93	41.85	37.77
303.15	7.11	7.11	27.95	10.25	41.72	36.66
308.15	7.17	6.79	32.41	15.68	40.82	34.23
313.15	7.62	6.83	28.06	9.97	40.34	32.75
318.15	7.73	6.80	35.00	22.00	39.57	30.46
323.15	6.94	5.12	26.23	6.86	39.05	29.10

#### Density modelling

Kumar^44–46^ proposed a simple method to predict the density of ternary solution, which can be expressed as follow

21 $$ \rho = \frac{{\left( {1 + \mathop \sum \nolimits_{j} m_{j} M_{j} } \right)}}{{\left\{ {\left( {\frac{1}{{d_{o} }}} \right)\left[ {\mathop \sum \nolimits_{j} y_{i} \left( {\left( {\frac{{d_{o} }}{{d_{j}^{o} }}} \right) - 1} \right) + 1} \right] + \mathop \sum \nolimits_{j} \frac{{m_{j} M_{j} }}{{d_{j}^{o} }}} \right\}}} $$

where *y*_*j*_ is the ionic strength fraction of the *j*th salt and can be written as

22 $$ y_{i} = \frac{{m_{o} }}{{m_{j}^{o} }} $$

where M is the molecular weight and *d* is the density. The subscript *j* is salt in the electrolyte solution and *d*_*o*_ is density of water.

**Figure 7:**
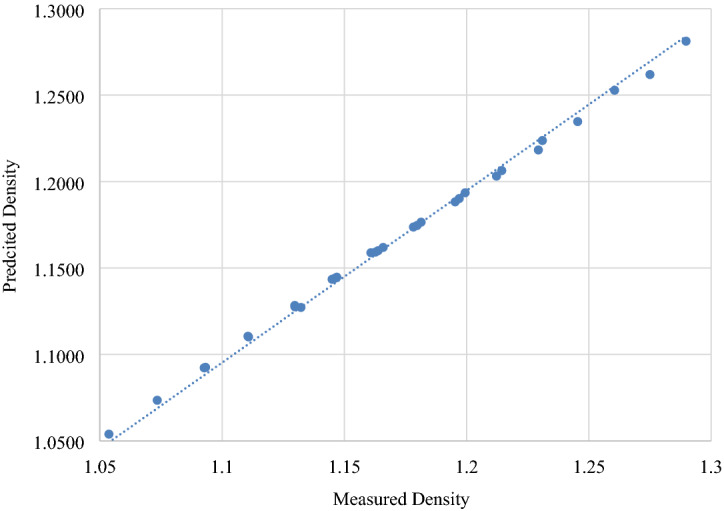
Plot of predicted values versus experimental data of density of ternary aqueous solution of KCl + CaCl_2_ + H_2_O at 323.15 K.

Equation (21) was used to predict the density of ternary solution at the molarities investigated in this work. Calculation method for Eq. (20) are available in the literature^44,45^. Density values calculated by Eq. (21) at all studied temperature are presented in Tables 3, 4, 5, 6, 7, 8 and 9. *AAD* and *SD* were also calculated and found to be s ranging from 0.12 to 0.40 and from 0.0018 to 0.0056 g cm^−3^ respectively. Experimental density and density predicted by Eq. (21) was plotted in Fig. 7 . Figure 7 and statistical calculations advocate that Eq. (21) proposed by Kumar is accurate enough to predict the density of the ternary solution for the concentrations and temperatures range used in this study. This is true for the NaCl + CaCl_2_ + H_2_O system as well^18^.

## Conclusions

Viscosity and density data for aqueous ternary system are still of interest for our industrial daily life applications. The measured data were compared with the available published data. The models used in this work were able to predict well the viscosity as function of both concentration and temperature and density of the specific system KCl + CaCl_2_ + H_2_O studied in this work. The Calculation method proposed in this work (Eq. 5) has been compared to other investigated ternary aqueous solutions investigated in previous published work^18^; NaCl + CaCl_2_ + H_2_O and NaCl + MgCl_2_ + H_2_O and reached the same conclusion.

The performance of the four models used in this work along with a representative plot of percentage deviations for all models is presented in Fig. 4. The exponential model is showing the least deviation because it is purely empirical and depends only on quality experimental ternary data. AAD for Hu model is also very low, although it is a predictive model because this model uses the empirically calculated A’s coefficients of a polynomial fitted to binary data. On the other hand, G-F model is showing little higher deviations than other models, as it is predictive model and depends on how accurately E and V values are calculated by fitting binary data to an equation based on absolute rate theory. Overall, performance of the model proposed in Eq. (5) is excellent. This model has the special characteristic of being associated with very fundamental Jones–Dole viscosity model. It is highly recommended to be used and validated for other ternary systems.
